# Maternal Effects on Anogenital Distance in a Wild Marmot Population

**DOI:** 10.1371/journal.pone.0092718

**Published:** 2014-03-20

**Authors:** Timothée D. Fouqueray, Daniel T. Blumstein, Raquel Monclús, Julien G. A. Martin

**Affiliations:** 1 Department of Ecology and Evolutionary Biology, University of California Los Angeles, Los Angeles, California, United States of America; 2 Département de Biologie, École Normale Supeérieure de Lyon, Lyon, France; 3 The Rocky Mountain Biological Laboratory, Crested Butte, Colorado, United States of America; 4 Departamento de Biología, Universidad Autónoma de Madrid, Madrid, Spain; 5 School of Biological Sciences, University of Aberdeen, Aberdeen, United Kingdom; University of Barcelona, Faculty of Biology, Spain

## Abstract

In mammals, prenatal exposure to sex steroid hormones may have profound effects on later behavior and fitness and have been reported under both laboratory and field conditions. Anogenital distance is a non-invasive measure of prenatal exposure to sex steroid hormones. While we know that intra-uterine position and litter sex ratio influence anogenital distance, there are other, heretofore unstudied, factors that could influence anogenital distance, including maternal effects. We capitalized on a long-term study of wild yellow-bellied marmots (*Marmota flaviventris*) to study the importance of maternal effects on explaining variation in anogenital distance and found significant effects. The strength of these effects varied annually. Taken together, our data highlights the strong variability due to environmental effects, and illustrates the importance of additive genetic and maternal genetic effects on neonatal anogenital distance. We suspect that, as others apply recently popularised quantitative genetic techniques to study free-living populations, such effects will be identified in other systems.

## Introduction

Maternal effects represent the influence of a mother's genotype or phenotype to her offspring's phenotype independently of additive genetic effects [1] and have numerous implications in a wide range of species on prenatal (development, immunity, stress level) and postnatal life (social rank, dispersal, reproductive performance, etc.) [2]–[5].

For mammals, the intrauterine environment is a potentially important source of maternal effects on a variety of life history traits [6]. Pre-natal exposure to sex steroid hormones emerges from the complex hormonal environment fetuses are exposed to. Each individual is exposed to androgens produced by its male siblings via transplacental and transamniotic diffusion [7]. Testosterone has organisational and activational effects on the central nervous system, masculinising and defeminising the behavior exhibited later in life [8], [9]. For instance, female rats (*Rattus norvegicus*) exposed to neonatal testosterone engaged in more masculinised impulsive behavior [9]. Similarly, masculinised yellow-bellied female marmots (*Marmota flaviventris*) had lower survival, were more likely to disperse, and had reduced weaning success compared with females exposed to reduced levels of intrauterine testosterone [10]. Thus, testosterone is an important modulator of life-history traits [10].

Apart from male siblings' testosterone, androgens are secreted by the mother's placenta, adrenal glands, and ovaries [11], [12], and they might contribute to offspring defeminisation or masculinisation. However, the importance of this maternal source has not been studied in the wild.

Early exposure to androgens is morphologically evident by the anogenital distance (AGD), the distance between the anus and the genital papilla. AGD results from the elongation of the perineal tissue, which is triggered by testosterone during the early development, and thus, males have larger AGD than females [13], [14]. AGD, at young ages, is thus a proxy of early exposure to androgens [15], [16] and is relatively easy to quantify under field conditions. Females (of several species) with larger AGD are masculinized. They are less likely to survive and more likely to disperse [10], are less likely to become pregnant [17], are less preferred by males, and have smaller and male-biased litters [18]. Similar findings have been observed as well in humans, including positive correlations between AGD and fertility in men [19], and ovarian follicles numbers in women [20].

We studied a population of individually marked wild yellow-bellied marmots for which litter effects but not maternal effects on anogenital distance were known: female yellow-bellied marmots' morphology, behavior, survival, and dispersal are influenced by the proportion of males in the litter [4], [10]. Given the strong relation between androgenization and life-history traits later in life, it seems crucial to disentangle the different sources of variation that might affect the offspring phenotype. For that we fitted an animal model, a statistical technique designed to decompose variance components, to assess how much of the variance in AGD in neonates was due to maternal, genetic and environmental effects.

## Methods

### Ethics Statement

Free-living yellow-bellied marmots were studied under research protocol ARC 2001-191-01 as well as permits issued by the Colorado Division of Wildlife. The research protocol was approved by the UCLA Animal Care Committee on 13 May 2002 and renewed annually. Trapping, measuring, and marking marmots are routine techniques that have been conducted with no deleterious consequences for over 50 years in this population of marmots. By design, marmots were not harmed during the course of this study.

### Experimental Subjects

Yellow-bellied marmots have been studied since 1962 in and around the Rocky Mountain Biological Laboratory, Gunnison County, CO, USA (elevation approx. 2890 m). Marmots are individually marked and followed throughout their lives. Natal emergence date was determined by daily observations of the colonies during the active season (mid-April to mid-September). As soon as they emerged from their natal burrow, pups were trapped, sexed, ear tagged, and fur marked with Nyanzol fur dye. Assigning marmots to sex is straightforward and was successfully repeated during numerous trapping sessions by different observers. Subjects were weighed using a digital scale (accurate to 25 g) and we measured the AGD using digital callipers (accuracy 1 mm). Every summer, new observers were trained to measure AGD accurately by making multiple AGD measurements on different individuals until they obtained measurements similar to trained observers. This training ensured consistency in our measurements across observers. Since all pups within a litter were not trapped and measured by the same observers, measurement error due to the observer should only increase the noise in the data and make it more difficult to detect significant effects.

Starting in 2002, we took a hair sample from every individual for DNA parentage assignment using 12 microsatellite loci. This permitted us to assign (with 95% confidence) paternity and maternity (for further details about the procedure and the study population, see [21]). The average litter size for yellow-bellied marmots is about 3–4 pups but varies from 1 to 10 pups. Females give birth at most once a year in the burrows, where the pups stay during lactation. They emerge about 25 days later to start foraging by themselves [22]. Litter sex-ratio was estimated using all pups trapped. For AGD analysis, however, we only used those individuals with full parentage and that were trapped within their first 10 days following natal emergence because AGD at that age is not as biased by morphological differences between individuals as it would be for older individuals [23]. We thus used AGD records from 564 pups, from 183 different litters, that were produced by 91 different mothers between 2002 and 2010. This represented 67% of all pups observed during that period.

### Statistical Analyses

Using the asreml function [24] in the statistical package R, v.2.14.1 [25], we fitted an animal model [26] to decompose the variance of AGD into its additive genetic, maternal (environment and genetic), litter and year components. An animal model is a particular type of mixed model in which the different individuals are not considered independent but related to each other by a matrix of relatedness (most often obtained from a pedigree) [26]. By fitting different random effects that are linked or not linked to a pedigree, it is thus possible to decompose the variance of a trait into its genetic and environmental effects. Random effects linked to the pedigree provide information of additive genetic variance whereas random effects not linked to the pedigree provide environmental variance estimates. Since an animal model is a mixed effect model, additional fixed effects could also be included to correct for potential biases in the variance estimates.

As fixed effects, we included mass (to correct for differences in body size), the number of days since emergence at trapping (to take into account the morphological development of the perineal tissue), sex (to control for sexual dimorphism), proportion of males in the litter (to control for litter effects), and litter size (to control for the number of siblings producing androgens). We tested the significance of the fixed effects using conditional Wald tests.

For random effects, we first fitted a model including only maternal identity (to estimate between mother variation), year (to assess the inter-annual environmental variation), and the identity of the litter. We then decomposed the maternal effects into its genetic and environmental components. Thus, we included pup identity linked to the pedigree (direct additive genetic effect), maternal identity (maternal environment), maternal identity linked to the pedigree (maternal genetic), year, and the identity of the litter. We tested the significance of random effects using a log-likelihood ratio test comparing the full model to a model without a specific random effect [27], [28]. Variance ratios for random effects were estimated using the estimated phenotypic variance from the animal model (i.e., the sum of variance parameters in the model after accounting for the fixed effects [28]).

## Results

As expected from previous analyses [10], we found a significant effect of litter sex ratio on AGD (Table 1), whereby pups in male-biased litters had greater AGDs. Male AGD was significantly greater than female AGD (Table 1) as previously [10], [29]. In addition, larger animals had larger AGDs (Table 1). For our sample of animals trapped within 10 days of emergence, there was no significant effect of the number of days since emergence, nor was there a significant effect of litter size (Table 1).

**Table 1:** Estimates (with standard error) of the fixed effects on anogenital distance of juvenile yellow-bellied marmots studied at the Rocky Mountain Biological Laboratory.

	Estimate (SE)	DF	F-cond	P
**(Intercept)**	4.899 (0.808)	1, 33.5	36.750	<0.001
**Sex [Male]** *	4.205 (0.185)	1, 468.1	514.300	<0.001
**Mass**	0.010 (0.001)	1, 388.7	124.100	<0.001
**Litter sex-ratio** †	1.749 (0.452)	1, 243.6	15.000	<0.001
Litter size	−0.058 (0.063)	1, 187.5	0.856	0.356
Days since emergence	−0.038 (0.038)	1, 453.7	0.963	0.327

Estimates significantly different from zero are in bold.

*: Females taken as reference

† : N males: N total

DF: numerator, denominator degrees of freedom

F-cond: conditional Wald F-test

**Figure 1:**
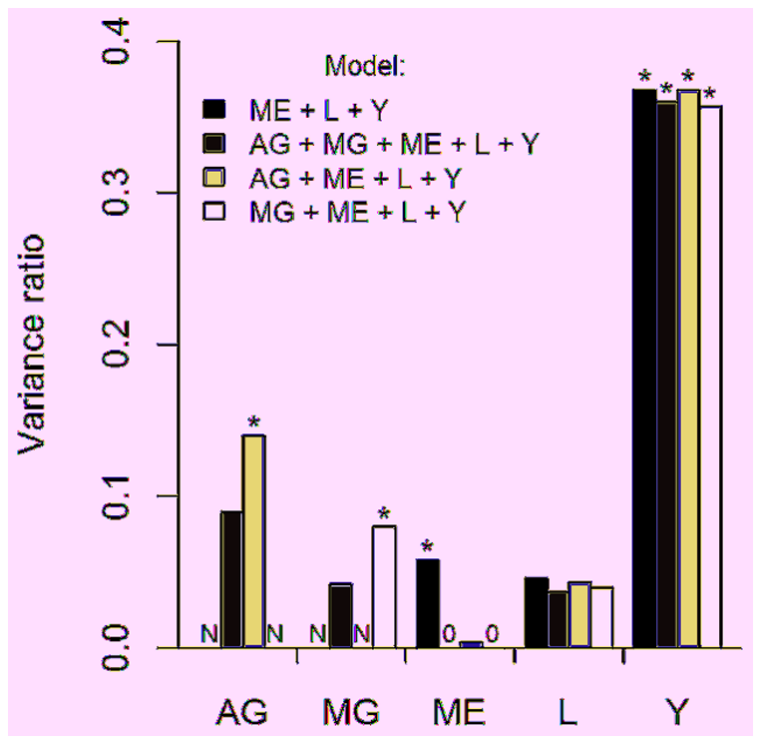
Variance ratio and components estimated for random effects from anogenital distance models of juvenile marmots. The random effects were additive genetic (AG), maternal genetic (MG), maternal environmental (ME), litter (L) and year (Y) effects. N indicates that an effect was not fitted in the model and a 0 indicates that the effect was fitted but estimated as zero. * indicates significant effects.

There were significant maternal effects at the phenotypic level accounting for 5.8% of the remaining variance (Fig. 1, Table 2, model 1, LRT  = 3.98, df  = 1, *P* = 0.046). When we decomposed the variance into additive genetic (LRT  = 1.51, df  = 1, *P* = 0.219), maternal environment (LRT  = 1.18, df  = 1, *P* = 0.277), and maternal genetic (LRT <0.001, df  = 1, *P*>0.999) components, we found that none of them were significant (Table 2, model 2). However, the inclusion of both additive genetic and maternal genetic effects provided a marginally better fit than when they were excluded (Fig. 1, Table 2, model 1 vs. model 2: *LRT*  = 5.56, df  = 2, *P* = 0.053). In addition, additive genetic (LRT  = 4.70, df  = 1, *P* = 0.030) and maternal genetic (LRT  = 4.39, df  = 1, *P* = 0.036) effects were both found to be significant if only one of them was included in the model and their estimates were larger (Fig. 1, Table 2, models 3 and 4). This suggests that despite nearly a decade's worth of data, we had insufficient power to conclusively isolate both effects. Nonetheless, it suggests that both effects could be important. When a maternal genetic effect was included in a model, the maternal environment explained <0.001 of the variance, a finding that suggests that most of the variance between mothers were due to maternal genetic effects. Litter identity effects were not significant in any model (Fig. 1, Table 2, all *P*>0.141). Year was significant in all models (all *P*<0.001) and explained 36% of the remaining variance in AGD (Fig. 1, Table 2).

**Table 2:** Estimates (with standard error) of variance components and proportion of variance of anogenital distance in yellow-bellied marmot pups.

		Variance component						Variance ratio				
Model	LL	AG	MG	ME	L	Y	P	AG	MG	ME	L	Y
1. **ME** + L + **Y**	−704.49	-	-	**0.390 (0.227)**	0.311 (0.236)	**2.480 (1.387)**	6.728 (1.447)	-	-	**0.058 (0.036)**	0.046 (0.036)	**0.368 (0.220)**
2. AG + MG +ME + L + **Y**	−701.56	0.613 (0.558)	0.287 (0.304)	0.000 (0.000)	0.252 (0.207)	**2.450 (1.372)**	6.800 (1.572)	0.090 (0.084)	0.042 (0.045)	0.000 (0.000)	0.037 (0.031)	**0.360 (0.218)**
3. **AG** +ME + L + **Y**	−702.14	**0.960 (0.518)**	-	0.029 (0.207)	0.300 (0.236)	**2.521 (1.407)**	6.847 (1.574)	**0.140 (0.082)**	-	0.004 (0.030)	0.043 (0.035)	**0.368 (0.222)**
4. **MG** +ME + L + **Y**	−702.32	-	**0.542 (0.260)**	0.000 (0.000)	0.274 (0.208)	**2.409 (1.348)**	6.742 (1.411)	-	**0.080 (0.042)**	0.000 (0.000)	0.040 (0.032)	**0.357 (0.213)**

AG: additive genetic; MG: maternal genetic; ME: maternal environment; L: litter; Y: year; P: phenotypic; LL: log-likelihood; Variance ratio of AG is the heritability, *h^2^*.

The raw phenotypic variance of AGD was 13.272. The phenotypic variance of AGD after controlling for the fixed effects was estimated as 6.743 (1.411). Significant random effects are in bold.

## Discussion

We have shown that 6% of the remaining variation in the AGD of marmot pups is explained by a maternal effect. Our results also suggest that AGD is heritable and influenced by maternal genetic and non-maternal environmental effects, thus indicating that a mother's genotype produces specific developmental conditions for her offspring. The presence of a maternal genetic effect on AGD could result from various mechanisms during gestation or lactation. It could be due to genetic differences in maternal care, maternal milk content or maternal stress. A more promising avenue of explanation might reside in utero during development. AGD is widely used as a proxy for prenatal androgen hormone exposure in rodents [14]. Prenatal exposure to androgens may influence vertebrate life-history traits [29]–[32], because organizational and activational events take place at this stage. This androgen-sensitive prenatal developmental window is thus a key period that may influence life-history traits. Here, a possible explanation of the maternal effects on the AGD would be such a hormonal transfer from the mother to her pups. However, some studies in laboratory rats, *Rattus norvegicus*, have found no correlation between maternal testosterone levels and fetal testosterone levels. However, they did find a negative relationship between maternal testosterone levels and the capacity of nutrient transport across the placenta, resulting in reduced fetal growth [33]. Therefore, maternal testosterone might indirectly affect offspring AGD by influencing the offspring mass. In primates, *in utero* exposure to androgens has been explored mainly through the study of the 2d:4d ratio. There is evidence that up to 66% of the variance is explained by additive genetic effects. In addition, there is evidence of strong environmental effects and for shared environment effects (including maternal environment and part of maternal genetic effects) [34]–[37]. Our results are thus in line with previous studies suggesting additive genetic, maternal environment and maternal genetic effects on *in utero* exposition to androgens. Nevertheless, further quantitative measurements of maternal and pup androgen levels, in addition to formal genetic analyses of maternal endocrinological traits are needed to formally evaluate the mechanism underlying the maternal genetic effect on AGD. Such studies, beyond the scope of our study of free-living animals, would greatly improve our understanding of the mechanistic origin of the maternal effect we identified.

While the existence of a significant litter composition fixed effect agrees with previous findings [10], it is noteworthy that the bulk of the remaining variation in AGD variance was explained by annual variation. Despite extensive training, personnel turnover between years could be responsible for some of this variation. However, other biologically relevant factors could also explain annual variation. For instance, the current environment (food availability, predation level, weather conditions) could also explain annual variation through its influence on maternal stress and body condition [4], [10].

In conclusion, AGD is a widely used proxy of early exposure to testosterone, and it is related to numerous other life-history traits [4], [10], [16], [38]. We showed that by decomposing sources of variation using the animal model, we were able to identify, for the first time in a wild population, both an additive genetic and a maternal genetic effect on AGD that is expected to also impact future life-history traits and fitness of the pups. When other researchers quantify the magnitude of maternal effects on AGD and its genetic basis, we will be in a better position to understand its general importance.
